# Designing Elastic Softening Under Compression via Dipole–Dipole Interactions: The Case of Na_2_TiSiO_5_

**DOI:** 10.1002/smll.74623

**Published:** 2026-07-29

**Authors:** Peijie Zhang, Alfonso Muñoz, Neha Bura, Pablo Botella, Catalin Popescu, Julio Pellicer‐Porres, Xiao Dong, Daniel Errandonea

**Affiliations:** ^1^ Departamento de Física Aplicada–Instituto de Ciencia de Materiales MALTA Consolider Team Universidad de Valencia Burjassot Valencia Spain; ^2^ Center For High Pressure Science and Technology Advanced Research (HPSTAR) Beijing China; ^3^ Departamento de Física MALTA Consolider Team Universidad de La Laguna Tenerife Spain; ^4^ CELLS–ALBA Synchrotron Light Facility Barcelona Spain; ^5^ Key Laboratory of Weak–Light Nonlinear Photonics School of Physics Nankai University Tianjin China

**Keywords:** bulk modulus, dipole‐dipole interaction, pressure‐induced softening

## Abstract

The bulk modulus (B), a key indicator of material compressibility, is generally expected to increase with pressure as interatomic repulsion intensifies. Conventional interatomic potentials are often approximated by nearly symmetric single‐well profiles. By contrast, dipole‐mediated interactions are directional and intrinsically asymmetric, allowing attractive interactions to strengthen during compression. This distinction suggests a chemical route to elastic softening that is not captured by conventional hardening models. Here, we identify Na_2_TiSiO_5_, synthetic paranatisite, as a model compound for pressure‐induced softening. Its bulk modulus *B*_0_ decreases sharply from 94(2) GPa (*Pmc*2_1_ phase) to 55.4(1.1) GPa (*Pca*2_1_ phase) above approximately 6.0 GPa. High‐pressure structural analyses reveal substantial shortening of the head‐to‐tail TiO_5_ alignment, while Born effective charge calculations indicate strengthened dipoles along the *c* axis. Consistently, Raman spectra show a pronounced redshift of the Ti–O stretching mode, indicating a lower Ti–O bond force constant. Together, these observations support a pressure‐driven rebalancing between Ti–O bonding and Ti–O···Ti dipolar interactions. Our results establish dipole‐interaction potentials as a design principle for compression‐softening materials and advance dipole‐driven elasticity engineering in polar solids.

## Graphical abstract

Pressure usually stiffens solids, yet Na_2_TiSiO_5_ exhibits anomalous elastic softening under compression. Unlike conventional mechanisms driven by geometric rearrangements or electronic spin‐state transitions, the softening originates from enhanced dipole coupling along head‐to‐tail TiO_5_ chains. Strengthened dipole–dipole interactions under pressure, therefore, provide an unconventional yet chemically intuitive mechanism for tuning elastic response in polar crystalline solids.

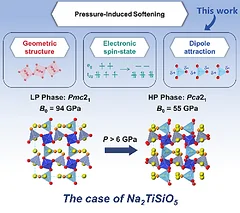

## 1 Introduction

**SCHEME 1:**
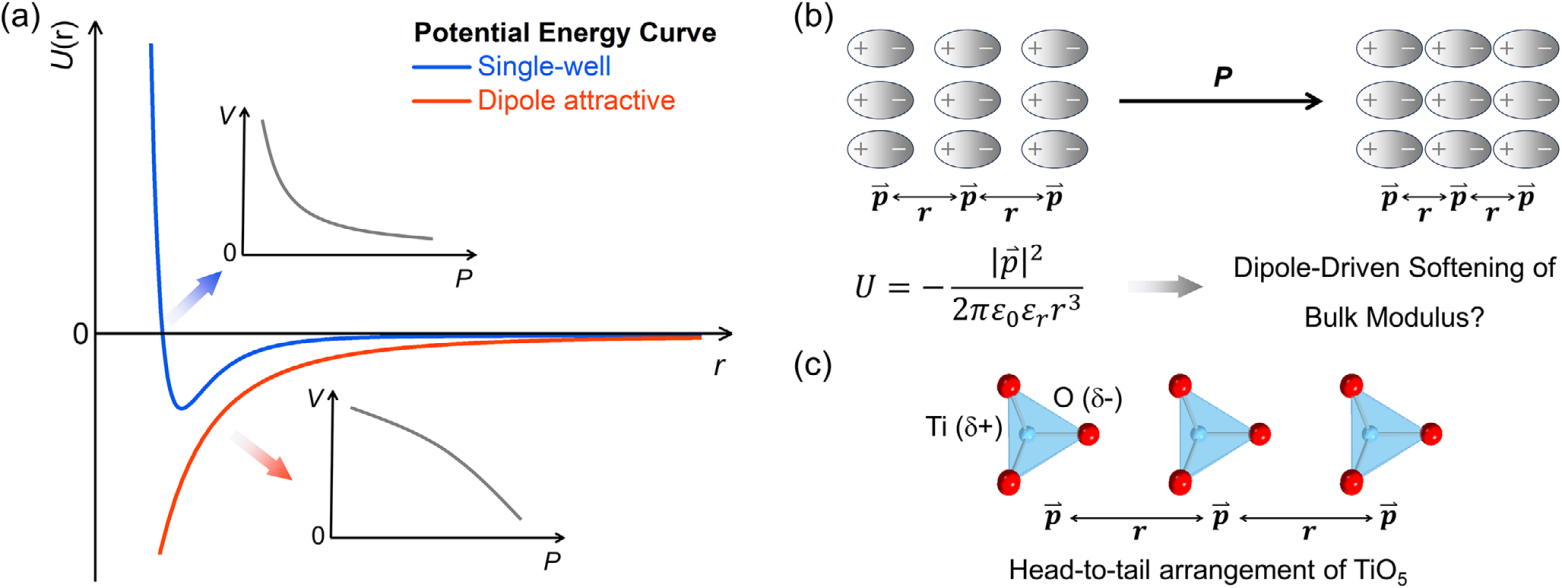
(a) Potential energy curves illustrating a single‐well potential (blue) and a dipole‐attractive potential (red) as a function of interatomic distance. Insets illustrate the corresponding relationship between volume *V* and pressure *P* under each potential. (b) Schematic of the hypothesis: in polar materials with head‐to‐tail dipoles, compression enhances dipole interactions and softens the bulk modulus. (c) Head‐to‐tail arrangement of TiO_5_ polyhedra, demonstrating the formation of a dipolar chain with Ti as the positive pole (δ^+^) and O as the negative pole (δ^−^).

The bulk modulus (*B*), the inverse of compressibility, quantifies a material's resistance to volume change under pressure and is widely used in geophysics and materials science to evaluate mineral behavior and mechanical response [1, 2, 3]. Conventional elastic responses are commonly described by approximately symmetric, single‐well interatomic potentials dominated by short‐range repulsion (Scheme 1a), which generally predict pressure‐induced stiffening regardless of whether the underlying interactions are covalent, ionic, or van der Waals in character [4, 5, 6]. Dipole–dipole interactions are fundamentally different. Their potentials are directional and asymmetric, and specific head‐to‐tail configurations can gain attractive stabilization as dipoles are brought closer (Scheme 1a) [7, 8, 9]. This feature suggests a possible route to pressure‐induced elastic softening, but direct experimental evidence for this mechanism remains lacking.

Here, we test the hypothesis that polar frameworks containing aligned head‐to‐tail dipolar units can soften under compression because enhanced dipole attractions offset conventional repulsive stiffening (Scheme 1b). Transition‐metal oxides provide a natural platform for this idea because metal *d*‐ligand *p* hybridization can stabilize off‐centered coordination environments and local electric dipoles. In ferroelectrics such as BaTiO_3_, for example, Ti–O hybridization is central to Ti displacement and polarization [10, 11, 12]. Low‐coordination TiO_5_ polyhedra are especially suitable because their asymmetric geometry can support oriented local dipoles and promote head‐to‐tail alignment, enabling dipole amplification under compression (Scheme 1c).

**FIGURE 1:**
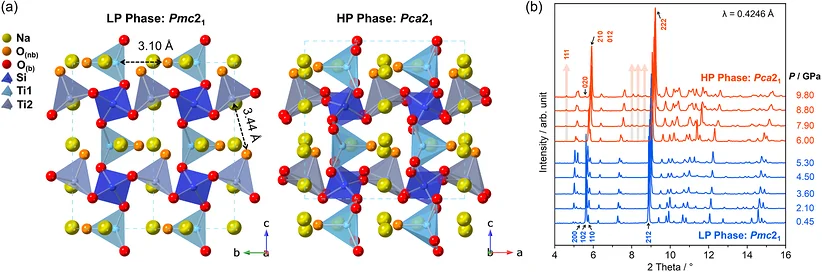
(a) Crystal structures of Na_2_TiSiO_5_: LP phase at ambient pressure and HP phase at 9.8 GPa. (b) Selected in situ powder XRD patterns of Na_2_TiSiO_5_ under high pressure.

To verify this hypothesis, we identified part structures containing head‐to‐tail TiO_5_ chains from The Materials Project (https://next‐gen.materialsproject.org), including Na_2_TiSiO_5_, Na_2_TiGe_2_O_5_, Sr_2_TiSi_2_O_8_, and BaTiSi_2_O_8_ (Figures 1a and S1). Among these candidates, Na_2_TiSiO_5_ was selected as a model system because it can be synthesized reproducibly and contains the targeted TiO_5_ dipolar‐chain motif. The broader applicability of the proposed mechanism to other identified frameworks remains a promising avenue for future experimental validation. Remarkably, Na_2_TiSiO_5_ exhibits an approximately 41% reduction in bulk modulus across the *Pmc*2_1_–*Pca*2_1_ phase transition, decreasing from 94(2) to 55.4(1.1) GPa. Our high‐pressure structural, vibrational, and charge analyses show that this softening arises from enhanced dipole–dipole coupling along the *c* axis, accompanied by rapid contraction of the nonbonded Ti···O distance and redshifting of the Ti–O stretching mode along this direction. These findings identify dipole coupling as a tunable mechanism for pressure‐dependent elasticity, extending compressibility control beyond conventional van der Waals and hydrogen‐bonded systems to materials governed by directional dipole–dipole interactions.

## 2 Results and Discussion

At ambient pressure, Na_2_TiSiO_5_ features TiO_5_ square pyramids connected via SiO_4_ tetrahedra, with Na^+^ in interstitial sites (Figure 1a). Each TiO_5_ unit has four bridged oxygens (O_(b)_, 1.9–2.0 Å) and one nonbridged oxygen (O_(nb)_, ∼1.7 Å), leading to stronger Ti *d*–O *p* overlap along O_(nb)_ and anisotropic charge distribution. This generates a pronounced dipole within each TiO_5_ unit. The polyhedra are arranged head‐to‐tail along *b*‐ and *c*‐axes, with Ti···O_(nb)_ distances of 3.10 and 3.44 Å, within the range where dipole–dipole interactions remain significant (*U*∝1/*r* ^3^) [13].

**FIGURE 2:**
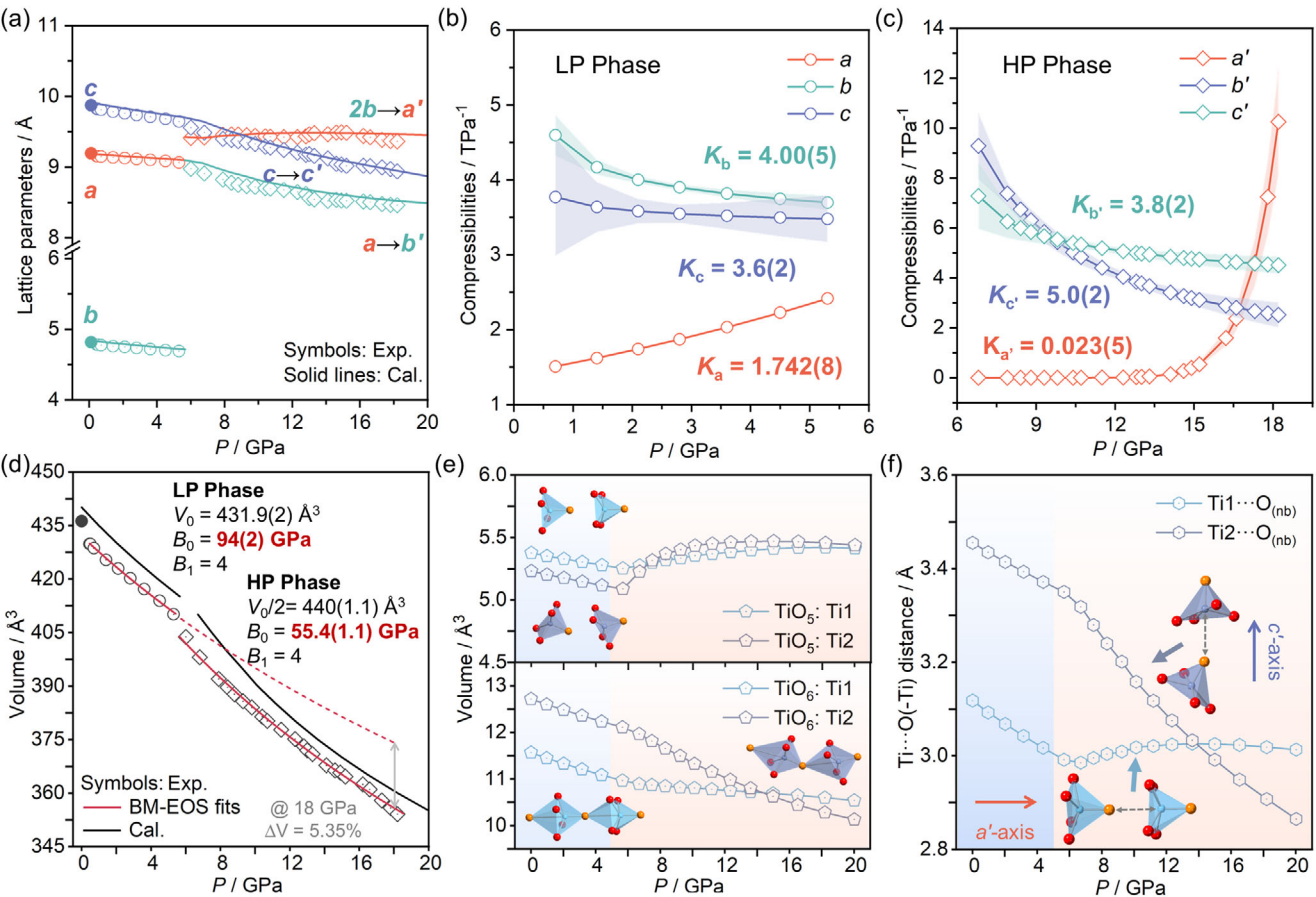
Pressure‐dependence of crystal structure evolution of Na_2_TiSiO_5_. (a) Unit cell parameters. Symbols: experimental results (solid: ambient pressure; hollow: high pressure), solid lines: DFT calculations. (b, c) Compressibility coefficients (*K*) of LP and HP phases, determined with PASCal [14]. The shaded regions denote the uncertainty bands. (d) Unit cell volume. Symbols: experimental results (solid: ambient pressure; hollow: high pressure), red solid lines: second‐order BM‐EOS fits, red dotted line: the extension of the LP phase BM‐EOS fit, and black lines: DFT calculations. (e) Volume of TiO_5_ square pyramids and schematically represented TiO_6_ octahedrons. (f) Ti···O_(nb)_(–Ti) nonbonded distance. All atomic analyses from DFT‐optimized structures.

In situ XRD measurements on Na_2_TiSiO_5_ were performed up to 19.9 GPa using Ne as the pressure medium (Figures 1b and S2). The orthorhombic low‐pressure (LP) phase *Pmc*2_1_ (*a* = 9.18 Å, *b* = 4.81 Å, and *c* = 9.86 Å) remains stable up to 5.3 GPa. Above 6.0 GPa, new peaks indicate a transition to a high‐pressure (HP) *Pca*2_1_ phase, in which the *b*‐axis doubles and *a*/*b* axes swap (Figure S3 and Table S1). The transition should be driven by distortion in TiO_5_ and SiO_4_ units, with SiO_4_ splitting into two distinct sites due to symmetry reduction (Figure S4). Above 13.0 GPa, only slight variations in diffraction peak intensities were observed, while the corresponding peak positions evolved continuously with pressure, suggesting the absence of an additional structural phase transition within the investigated pressure range (Figure S5). As shown in Figure 2a, lattice parameters from Le Bail fits match well with density functional theory (DFT) calculations. To explore unit cell behavior, compressibility coefficients (*K*) were calculated (Figure 2b,c). Interestingly, contraction behaviors differ between phases: in the LP phase, the *b*‐ and *c*‐axes dominate compression, while in the HP phase, contraction shifts to the *b*′‐ and *c*′‐axes (equivalent to the LP *a*‐ and *c*‐axes). The compressibility coefficients for the HP *b*′‐ and *c*′‐axes (3.8(2) and 5.0(2)) are higher than those in the LP phase (1.742(8) and 3.6(2)). Regarding the anisotropic compressibility, a detailed structural explanation is provided following Figure S6. Pressure‐volume data fitted with second‐order Birch–Murnaghan equations of state (BM‐EOS) yields *B*_0_ = 94(2) GPa for LP and 55.4(1.1) GPa for HP, revealing enhanced compressibility in the HP phase [6]. To further evaluate the elastic evolution within the HP phase, third‐order BM‐EOS fitting was also performed (Figure S7). The resulting pressure derivative of the bulk modulus is *B*_1_ ≈ 4.1, close to a conventional value, indicating that the HP phase itself does not exhibit continuous softening upon further compression. Therefore, the softening discussed in this work refers to the substantial reduction in the initial bulk modulus across the LP–HP structural transition, rather than anomalous pressure evolution within the HP phase itself. At 18 GPa, the HP phase has 5.35% lower volume without a clear collapse, consistent with DFT results. Furthermore, our combined experimental and theoretical results indicate that the phase transition may occur near a weakly first‐order transition or a tricritical point, where continuous symmetry breaking is accompanied by discontinuities in the elastic response functions (Figure 2b–d) [15, 16].

To confirm the phase transition from an energetic perspective, the enthalpy difference (Δ*H*) between the HP *Pca*2_1_ and LP *Pmc*2_1_ phases was calculated. The HP phase becomes energetically favorable above 5.6 GPa, consistent with experimental observations (Figure S8). In addition, thermodynamically, the enthalpy change is mainly affected by the Δ*PV* term, which means that the Δ*H* difference also could be estimated based on the formula: Δ*H* ≈ *P*[*V*_0, HP_(1‐*P*/*B*_0, HP_)‐*V*_0, LP_(1‐*P*/*B*_0, LP_)]. This result is consistent with the DFT calculation, which verifies the rationality of the *B*_0_ and *V*_0_ obtained in the HP‐ XRD experiment.

To understand the reduced bulk modulus in the HP phase, pressure‐dependent volumes of individual polyhedra were analyzed (Figures S9 and 2e). The volumes of SiO_4_ tetrahedron and NaO_6_ octahedron undergo a normal decrease with increasing pressure, which excludes their conventional contribution to the compressibility enhancement. The volumes of TiO_5_ square pyramids do not decrease after the phase transition but increase abnormally, which also cannot explain the decrease of the bulk modulus. However, if TiO_5_ square pyramids arranged head to tail are schematically represented as TiO_6_ octahedron along the *a*′‐axis for Ti1 and *c*′‐axis for Ti2, respectively, the volume of TiO_6_ octahedron for Ti2 shrinks faster after the phase transition, which corresponds to the maximum compressibility coefficient (*K*_c_ = 5.0(2)) of the *c*′‐axis in the HP phase (Figure 2c,e). This prompts a detailed analysis of the pressure‐dependent Ti–O lengths and the distance between the O_(nb)_ and the next adjacent Ti. Compared with the average bond length of Ti–O_(b)_ bond, which shows a significant decrease with increasing pressure, the bond length of Ti–O_(nb)_ bond remains almost unchanged with increasing pressure (Figure S10). This is because the Ti–O_(b)_, being part of the structural framework, has greater flexibility to accommodate external pressure through bond length adjustments. Whereas the Ti–O_(nb)_ bond, being terminal, exhibits less variation under pressure because the O_(nb)_ is not shared between polyhedra and its position reflects the Ti–O equilibrium bond length instead of a result of a compromise between competing bond length requirements. The changes in Ti–O bond lengths still indicate that they are not responsible for the anomalous decrease in compressibility. As shown in Figure 2f, the pressure‐dependent distance between Ti2 and O_(nb)_ in the next TiO_5_ clearly shows a sharp decrease after the phase transition, which is undoubtedly responsible for the reduction in the volume of TiO_6_ octahedron for Ti2. While even at 20 GPa, the distance between Ti2 and O_(nb)_ is still larger than 2.80 Å, far greater than the chemical bond length range of the Ti–O [17]. Therefore, the coordination number of the titanium oxide polyhedron remains unchanged upon compression up to 20 GPa. This is further confirmed in the analysis of the electron localization function (ELF). The ELF distribution at 20 GPa shows that there is a lack of significant electron cloud overlap signals between the TiO_5_ square pyramids (Figure S11), indicating that the interactions between these polyhedrons are weak [18]. Therefore, the hypothesis of polyhedral transformation from TiO_5_ square to TiO_6_ octahedron under high pressure can be ruled out. This conclusion supports the stability of TiO_5_ square pyramids as basic structural units and suggests that weak dipole–dipole interactions arising from the polar nature of the Ti–O bonds may dominate the fine‐tuning of the crystal structure, rather than the direct transformation of the polyhedron.

Elastic constants of Na_2_TiSiO_5_ were calculated for the LP phase at 0 GPa and HP phase at 12.3 GPa (Table S2), confirming mechanical stability via the Born criteria [19]. Using the Hill approximation, the calculated mechanical properties are given in Table 1 [20]. The LP phase has *B* = 82.78 GPa, *G* = 49.64 GPa, and *E* = 124.11 GPa, which indicate a stiff, brittle framework (Poisson's ratio *ν* = 0.25, Pugh's ratio B/G = 1.67). In the HP phase, *B* remains nearly unchanged at 84.08 GPa, implying *B*_0_ ≈ 34.88 GPa when *B*_1_ = 4 is decreased and consistent with the trend of the *B*_0_ reduction in Na_2_TiSiO_5_ at high pressure (experimental results: *B*_0_ = 94(2) GPa for LP phase and *B*_0_ = 55.4(1.1) GPa for HP phase). The lower DFT‐derived bulk modulus of the HP phase relative to experiment may originate from some intrinsic limitations of DFT for framework compressibility, such as the exchange‐correlation functional dependence and the absence of finite‐temperature effects. Nevertheless, the calculations correctly reproduce the qualitative compressional behavior of the HP phase, including its relatively reduced bulk modulus. Furthermore, shear modulus drops to *G* = 36.21 GPa and *E* = 94.98 GPa, while *ν* increases to 0.31 and B/G rises to 2.32, exceeding the ductility threshold (1.75). These changes indicate pressure‐induced softening in shear resistance and a mechanical transition from brittle to ductile behavior under high pressure.

**TABLE 1:** The calculated bulk (*B*), shear (*G*), and Young (*E*) moduli, Poisson ratio (*ν*), *B*/*G* ratio of Na_2_TiSiO_5_.

Properties	LP phase (0 GPa)	HP phase (12.3 GPa)
*B* (GPa)	82.78	84.08
*B*_0_ (GPa) if *B*_1_ = 4	82.78	34.88
*G* (GPa)	49.64	36.21
*E* (GPa)	124.11	94.98
*ν*	0.25	0.31
B/G	1.67	2.32

**FIGURE 3:**
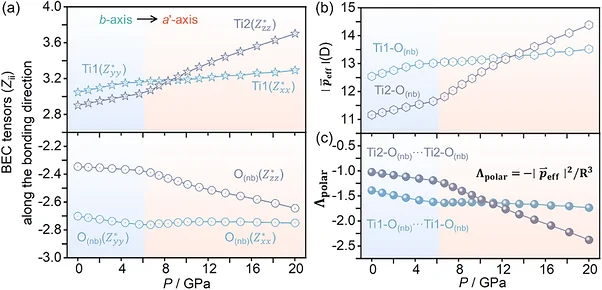
(a) Pressure‐dependence of BEC tensors of Ti and O_(nb)_ atoms along the crystal axes corresponding to the bonding direction. (b) Pressure‐dependent |${\overset{⇀}{p}}_{\mathrm{eff}}$| of Ti‐O_(nb)_ bonds. (c) Pressure‐dependent Λ_polar_ of dipole–dipole interaction.

The directional compressibility of a crystal reflects changes in the curvature of its atomic potential energy surface, with higher compressibility corresponding to reduced curvature along that direction. In Na_2_TiSiO_5_, head‐to‐tail TiO_5_ pyramids may create attractive dipole‐dipole interactions, lowering the energy cost for atomic displacements. To quantify this, the Born effective charge (BEC) tensors $Z_{\alpha ,\;ij}^{*}$= $\frac{\partial P_{i}}{\partial u_{\alpha ,j}}$ were calculated from 0 to 20 GPa (Table S3) [21, 22]. Here, the BEC tensors are treated as effective descriptors of local polarization response rather than formal static ionic charges, consistent with the modern theory of polarization and previous studies on polar oxides and ferroelectrics [23, 24, 25]. Focus was placed on $Z_{yy}^{*}$ (equivalent HP $Z_{xx}^{*}$) for Ti1–O_(nb)_, and $Z_{zz}^{*}$ for Ti2–O_(nb)_. At ambient pressure, $Z_{yy}^{*}$ of Ti1 and $Z_{zz}^{*}$ of Ti2 are +3.05 and +2.90, smaller than perpendicular directions (Ti1: $Z_{xx}^{*}$= +4.65, $Z_{zz}^{*}$= +4.76; Ti2: $Z_{xx}^{*}$= +4.64, $Z_{yy}^{*}$= +4.52) due to localized electron density along Ti–O_(nb)_ bonds. With increasing pressure, $Z_{zz}^{*}$ of the Ti2 and O_(nb)_ rise sharply (28% and 12% at 20 GPa), whereas changes in Ti1–O(nb) are <8% and <2%; BECs perpendicular to the bond remain nearly unchanged (Figures 3a and S12). Such anisotropic enhancement of the BEC response indicates increasing polarization sensitivity along the Ti2–O(nb) direction under compression, likely associated with pressure‐induced modulation of Ti–O hybridization and local bonding geometry.

The effective local polarization descriptor, defined as the effective polarization dipole ${\overset{⇀}{p}}_{\mathrm{eff}}$ of Ti–O_(nb)_ bonds, was constructed (Tables S4 and S5). In the LP phase, |${\overset{⇀}{p}}_{\mathrm{eff}}$| grows slowly, but after the phase transition, |${\overset{⇀}{p}}_{\mathrm{eff}}$| of Ti2–O(nb) increases sharply, consistent with BEC trends (Figure 3b). Correspondingly, the phenomenological polar‐coupling parameter $\Lambda_{\mathrm{polar}}=-|{\overset{⇀}{p}}_{\mathrm{eff}}{|}^{2}/R^{3}$ along *c*‐axis increases by 132% at 20 GPa (Figure 3c and Table S6), driven jointly by the contraction of the *c*‐axis and the enhancement of the Ti2–O_(nb)_ local polar response. This behavior suggests a positive‐feedback mechanism of polar‐lattice coupling: enhanced local polarization interactions promote further shortening of the Ti2–O_(nb)_···Ti2–O_(nb)_ separation along the *c*‐axis, while the reduced inter‐unit distance further strengthens the effective polar coupling. Such cooperative interactions lower the energetic cost of compression and contribute to the anomalous reduction of the bulk modulus in the HP phase. In contrast, the Λ_polar_ associated with the Ti_1_–O(nb) units only increases by <21% up to 20 GPa.

To further assess the origin of dipole behavior, Bader charge analysis was also performed (Figure S13). The minor and non‐monotonic evolution of the Bader charges under pressure indicates that the observed dipolar behavior does not originate from substantial static charge transfer between atomic species. Instead, the electronic charge distribution remains largely conserved during compression, suggesting that the pressure‐induced polarity is dominated by anisotropic electronic polarization rather than conventional ionic charge separation.

**FIGURE 4:**
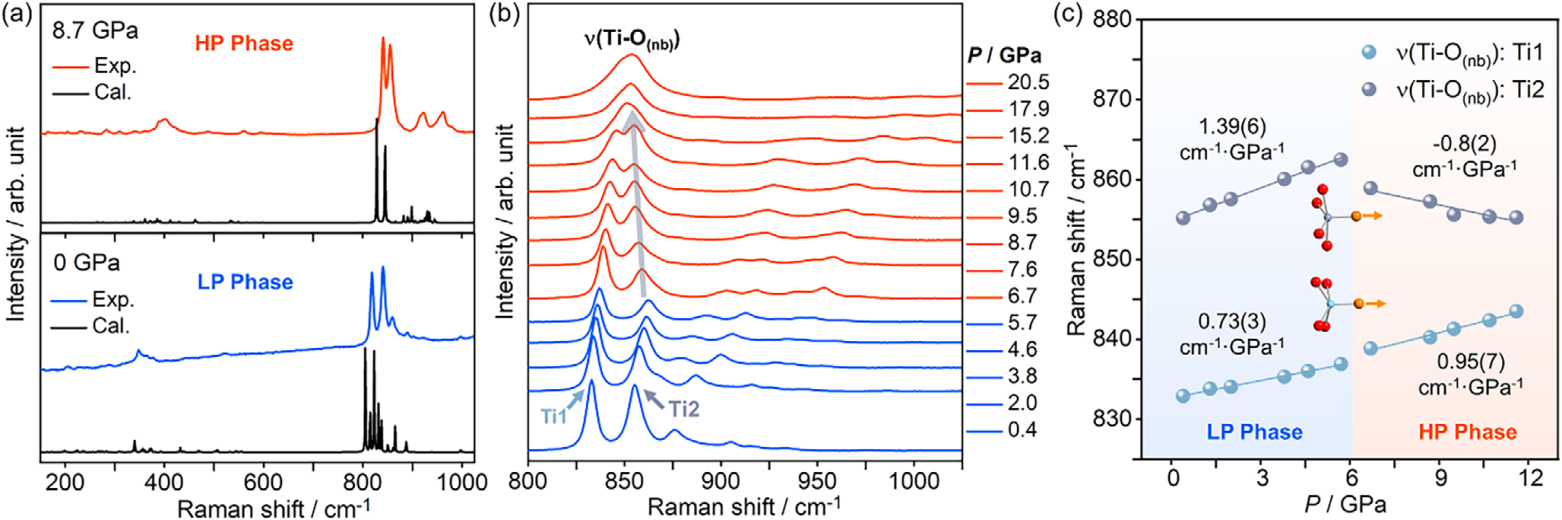
(a) Raman spectra of the LP phase and the HP phase of Na_2_TiSiO_5_ from the experiment and calculation. (b) Selected in situ Raman spectra in the range between 800 and 1025 cm^−1^. (c) Pressure‐dependence of the Ti–O_(nb)_ stretching mode. The symbols represent the data obtained from experiments, and the solid lines are the theoretical fit to it. The resulting slope values are mentioned with each fitting.

To track bond evolution under pressure, in situ Raman spectroscopy was performed with 633 nm excitation (Figures 4 and S14). The calculated spectrum of the *Pmc*2_1_ phase at 0 GPa reproduces experimental features well (Figure 4a). Two prominent modes observed between 825 and 875 cm^−1^ are attributed to the *ν*(Ti–O(nb)) stretching vibrations, in agreement with the calculated Raman modes (Figure S15). They originate from displacements of the nonbridging O atoms, and their high Raman intensity indicates substantial polarizability variations associated with the asymmetric charge distribution of the Ti–O_(nb)_ bond [23]. Above 6.7 GPa, the *Pca*2_1_ phase generates new features (e.g., 950 cm^−1^), confirmed by calculations (Figures 4a and S16). The two *ν*(Ti–O(nb)) modes show distinct pressure trends: Ti1‐centered vibrations continuously blueshift (0.73(3) and 0.95(7) cm^−1^·GPa^−1^ in the LP and the HP regions), indicating bond stiffening, while the Ti2‐associated mode redshifts in the HP phase (−0.8(2) cm^−1^·GPa^−1^), signifying force constant reduction and bond softening (Figure 4b,c). Moreover, quantitative mode Grüneisen parameters (*γ*) show a negative *γ* (−0.08) for the Ti2–O_(nb)_ mode in the HP phase, while Ti1–O_(nb)_ modes remain positive (0.1) (Table S7). Above 15.2 GPa, the modes merge due to peak convergence and broadening. Notably, the Ti2–O_(nb)_ bond does not exhibit the type of anomalous bond elongation typically associated with stretching‐mode softening under compression, suggesting that the observed Raman redshift cannot be explained by geometric effects. With geometric effects ruled out, the redshift can be directly associated with dipole–dipole interactions along Ti–O_(nb)_···Ti chains. Ti2‐centered dipoles strengthen in the HP phase, lowering the local curvature of the potential energy surface, reducing force constants, and producing Raman redshift, consistent with pressure‐induced softening inferred earlier. In contrast, Ti_1_ dipoles are weak and follow conventional stiffening (Figure 3b,c). These results provide direct spectroscopic evidence that the anomalous bulk modulus reduction in Na_2_TiSiO_5_ arises from dipole–dipole interactions. Furthermore, the Raman spectrum collected after decompression was nearly identical to that of the pristine sample, indicating that the pressure‐induced phase transition is reversible (Figure S17).

The simultaneous contraction of the Ti2···O_(nb)_ distance and redshift of the Ti2‐associated *ν*(Ti–O_(nb)_) mode recalls a pressure response widely reported for hydrogen‐bonded X–H···Y systems (Figures 2f, 4c, and S18). In these systems, compression shortens the nonbonded H···Y contact, while the X–H stretching mode often redshifts because electronic redistribution weakens the effective restoring force of the X–H bond [24, 25, 26]. A similar phenomenon is observed in the Ti–O···Ti arrangement, suggesting a rebalancing of nonbonded interactions (*e.g*., H–bond and dipole interactions) and chemical bonding under high pressure. Such local rebalancing may also influence macroscopic elasticity during compression. A related example is ice, which shows anomalous elastic softening between 42.4 and 54 GPa, followed by rapid hardening at higher pressures [26].

Combining HP‐XRD, BEC calculations, and HP‐Raman spectroscopy, we establish a coherent mechanism for pressure‐induced elastic softening in Na_2_TiSiO_5_. Rather than following the conventional repulsion‐dominated stiffening response, compression amplifies the head‐to‐tail dipolar interaction associated with the local Ti–O···Ti configuration. HP‐XRD captures both the macroscopic softening and its structural origin, namely, the rapid contraction of the non‐bonded Ti2···O_(nb)_ separation along the *c* direction. Complementary BEC calculations show that compression enhances the effective charge response along the same direction, supporting a strengthened local dipole contribution. Consistently, HP‐Raman spectra reveal that Ti2···O_(nb)_ contraction is accompanied by a redshift of the Ti2‐associated *ν*(Ti–O_(nb)_) mode, indicating a reduction in the Ti–O bond force constant. Together, these observations show that compression does not merely stiffen the lattice through enhanced short‐range repulsion. Instead, it can rebalance local electrostatic interactions and chemical bonding, weakening the effective restoring force along a specific structural direction and producing macroscopic elastic softening. This work therefore, provides an experimental framework for identifying dipole‐mediated softening under pressure. Broader comparative studies across related dipolar and non‐dipolar systems will be essential to determine how general this mechanism is and which structural features best promote it.

**FIGURE 5:**
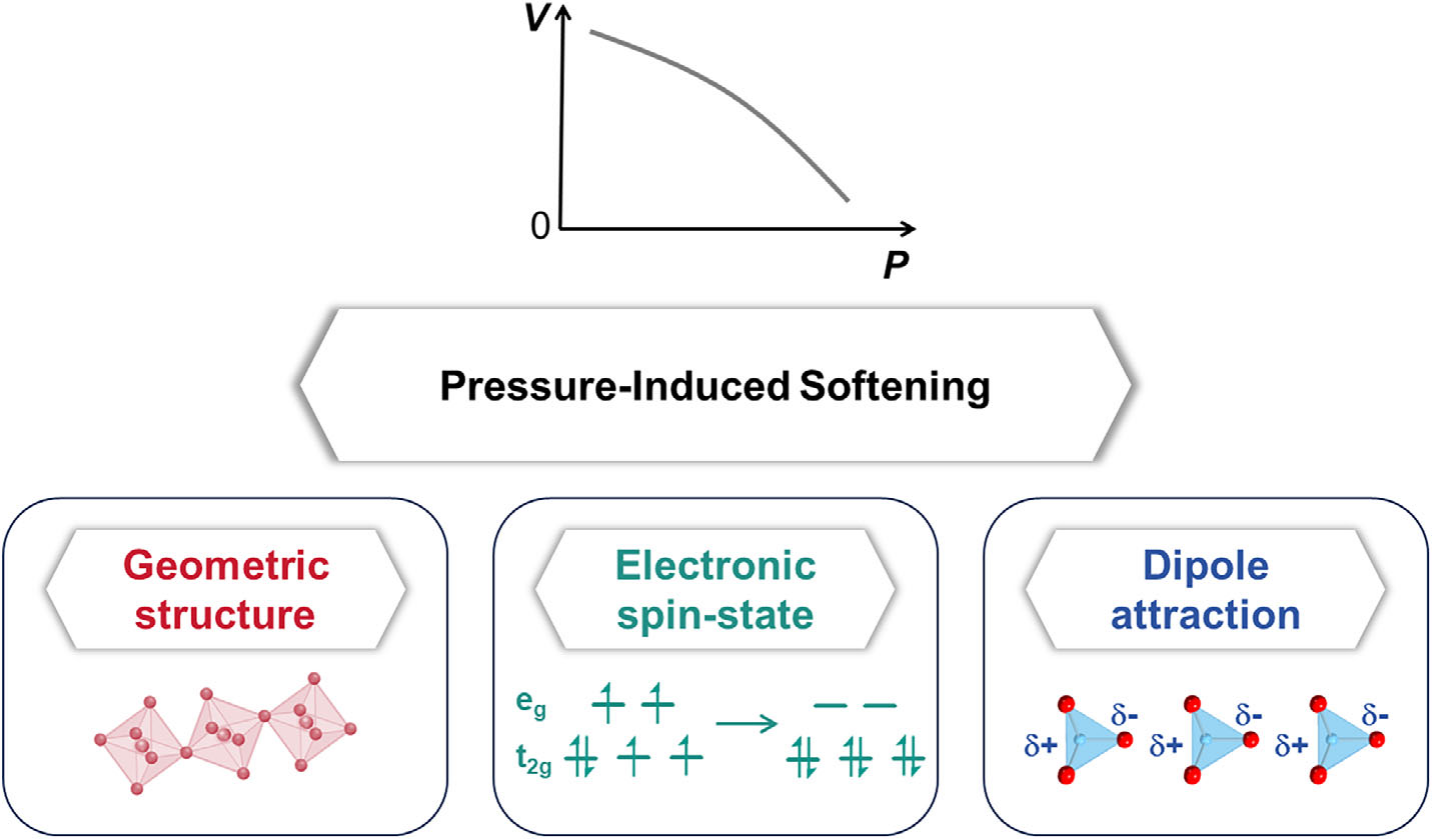
Summary of the mechanisms of pressure‐induced softening.

Previously reported pressure‐induced softening mechanisms typically involve two processes (Figure 5). The first is governed by structural geometric degrees of freedom, such as framework hinging or rigid‐unit rotations [27]. This mechanism relies on low‐energy structural modes, allowing the lattice to accommodate compression via angular distortions rather than bond shortening, leading to softening without significant electronic reorganization. In polyhedral networks, compression is facilitated if units can buckle or rotate without distorting individual bonds, as buckling absorbs stress at lower energy than direct bond compression. Such behavior occurs in amorphous silica [28, 29], and negative thermal expansion materials like ZrW_2_O_8_ and Zn(CN)_2_ [30, 31], showing that structural flexibility can induce anomalous elastic responses under pressure. The second mechanism involves electronic spin‐state transitions, where pressure induces high‐spin to low‐spin transformations in transition‐metal ions [32], causing abrupt changes in ionic radii and local bonding, and often resulting in anomalous elasticity. This has been observed in (Mg, Fe)O ferropericlase [33, 34, 35], which is crucial for understanding large variations in seismic *P*‐velocities and viscosity [36, 37, 38].

The third, fundamentally distinct, mechanism in this system is pressure‐enhanced dipole–dipole attraction between polarized units (Figure 5). Unlike the first two mechanisms, this softening arises from amplification of long‐range electrostatic interactions under compression. As pressure increases, attractive dipole–dipole forces grow faster than short‐range repulsion, causing unconventional lattice softening. Our results highlight that both local chemical environments and long‐range interactions influence pressure‐responsive mechanics. Dipole‐active units, such as TiO_5_ polyhedra, act as chemically tunable elements whose local bonding polarity couples to macroscopic compressibility. While demonstrated for Na_2_TiSiO_5_, this mechanism is general: many ferric oxides, framework silicates, organic frameworks, and hybrid materials host intrinsic dipoles and can exhibit similar anomalies. Notably, previous HP experiments on Na_2_TiGeO_5_ also reported an anomalous reduction of the bulk modulus across a pressure‐induced phase transition [39]. Our reanalysis shows its bulk modulus decreased from 89(2) GPa in the LP phase to 43(6) GPa in the HP phase (Figure S19). Although the original study could not establish the microscopic structural origin of this behavior due to the limitations of energy‐dispersive XRD and second‐generation synchrotron available at that time, the observed elastic softening is qualitatively consistent with the mechanism proposed here. Our results thus provide a predictive design principle for piezoelectric and dielectric materials, offer alternative interpretations for seismic or elastic anomalies, and inform stress‐strain engineering in multifunctional oxides.

However, all pressure‐induced softening mechanisms are inherently limited by the fundamental increase of short‐range interatomic repulsion at sufficiently high pressures. Ultimately, no softening mechanism can persist indefinitely. With continued compression, the intrinsic stiffness of chemical bonds and the rapidly increasing short‐range repulsion become dominant, leading to a recovery and subsequent increase of the bulk modulus [26]. This behavior reflects the general tendency of condensed matter to stiffen at sufficiently high pressures. Nevertheless, the existence of anomalous softening regimes demonstrates that, within well‐defined physical constraints, specific structural and electronic factors can significantly delay or counteract this stiffening over a finite pressure range.

## 3 Conclusion

In summary, we reveal a previously unrecognized mechanism of pressure‐induced softening in Na_2_TiSiO_5_, arising from an unusual potential energy curve dominated by attractive dipole interactions. This effect is marked by a dramatic decrease in *B*_0_–from 94(2) GPa in the LP phase to 55.4(1.1) GPa in the HP phase above ∼6.0 GPa–an unprecedented drop that defies conventional expectations of stiffening under compression. Structural, BEC, and vibrational analyses collectively confirm that the head‐to‐tail alignment of TiO_5_ square pyramids strengthens dipole–dipole attraction under pressure, producing a pronounced *c*‐axis contraction and driving the bulk‐modulus softening. These results identify dipolar attraction as a critical yet previously overlooked mechanism for mechanical tuning in polar framework materials. More broadly, they establish dipole interactions as an important design parameter for achieving pressure‐responsive elasticity in polar solids.

## Conflicts of Interest

The authors declare no conflicts of interest.

## Supporting information




**Supporting File 1**: smll74623‐sup‐0001‐SuppMat.docx.

**Supporting File 2**: smll74623‐sup‐0002‐cif.zip.

## Data Availability

The data that support the findings of this study are available from the corresponding author upon reasonable request.
