# Supplementary material for: Designing Elastic Softening Under Compression via Dipole–Dipole Interactions: The Case of Na2TiSiO5
Source: Small. 2026 Jul 29;22(51):e74623. doi: 10.1002/smll.74623 (PMC13569043; doi:10.1002/smll.74623)
Supplement: Supplementary file 1 [file SMLL-22-e74623-s001.docx]

Supporting Information

**Designing Elastic Softening under Compression via Dipole–Dipole Interactions: The Case of Na_2_TiSiO_5_**

Peijie Zhang*, Alfonso Muñoz, Neha Bura, Pablo Botella, Catalin Popescu, Julio Pellicer–Porres, Xiao Dong, Daniel Errandonea

**Abstract:** The bulk modulus (*B*), a key indicator of material compressibility, is generally expected to increase with pressure as interatomic repulsion intensifies. Conventional interatomic potentials are often approximated by nearly symmetric single-well profiles. By contrast, dipole-mediated interactions are directional and intrinsically asymmetric, allowing attractive interactions to strengthen during compression. This distinction suggests a chemical route to elastic softening that is not captured by conventional hardening models. Here we identify Na_2_TiSiO_5_,synthetic paranatisite, as a model compound for pressure–induced softening. Its bulk modulus *B*_0_ decreases sharply from 94(2) GPa (*Pmc*2_1_ phase) to 55.4(1.1) GPa (*Pca*2_1_ phase) above approximately 6.0 GPa. High-pressure structural analyses reveal substantial shortening of the head-to-tail TiO_5_ alignment, while Born effective charge calculations indicate strengthened dipoles along the *c* axis. Consistently, Raman spectra show a pronounced redshift of the Ti–O stretching mode, indicating a lower Ti–O bond force constant. Together, these observations support a pressure-driven rebalancing between Ti–O bonding and Ti–O···Ti dipolar interactions. Our results establish dipole-interaction potentials as a design principle for compression-softening materials and advance dipole-driven elasticity engineering in polar solids.

Experimental Procedures

**Methods**

*Synthesis of Na_2_TiSiO_5_*: The polycrystalline sample of Na_2_TiSiO_5_ was prepared by a two-step process which involves hydrothermal reaction followed by high temperature reaction. A gel prepared from the nominal composition of 5.6 Na_2_O: 3.1 SiO_2_: 1 TiO_2_: 4 Isopropanol: 130.4 H_2_O (all in molar ratio) was aged for 12h and then filled into an autoclave. The autoclave was heated at 230°C for 3days and then cooled to room temperature by putting off the furnace. The product was separated and washed thoroughly with demineralized water until the pH of the washing solution became constant. The powder was dried at ambient temperature and then heated to 950°C for 3h in static air environment using a platinum sample holder. The obtained sample was characterized by Rietveld refinement of powder XRD. The XRD pattern agrees well with the reported structural data (82153, 259048-ICSD).^[40-41]^

*In situ high-pressure XRD and Raman experiments*: HP powder XRD measurements were performed using a membrane-driven diamond anvil cell (DAC) equipped with 500 μm culet diamonds. An Inconel gasket, initially 200 μm thick, was pre-indented to 50 μm, and a 250 μm diameter hole was drilled at its center to form the sample chamber. Neon (Ne) was employed as the pressure-transmitting medium to ensure quasi-hydrostatic conditions in both the high-pressure XRD and Raman experiments. Using Ne as the pressure-transmitting medium provides a nearly hydrostatic pressure environment up to at least 15 GPa, while the deviatoric stress remains negligible even at the maximum pressure investigated here (20 GPa).^[42]^ A copper (Cu) grain was co-loaded with the sample to serve as an internal pressure calibrant, with pressure determined from the Cu (111) reflection using its known EOS.^[43]^ The experiments were conducted at the MSPD beamline of the ALBA synchrotron using a monochromatic beam of wavelength 0.4246 Å.^[44]^ XRD patterns were collected on a Rayonix charge-coupled device detector and LaB_6_ was applied to calibrate the geometry parameters. Dioptas software was used to reduce the collected data.^[45]^ Refinement and indexation were performed using Jana 2006 software, Material Studio, and PowderCell.^[46-47]^ HP Raman experiments of powder samples were performed at room temperature using the DAC with ultra-low fluorescence and Ne as a PTM. Raman spectra were collected in the backscattering geometry using a 633 nm He-Ne laser and a Jobin-Yvon spectrometer in combination with a thermoelectric-cooled multichannel CCD detector with spectral resolution around 2 cm^-1^. The pressure was measured by the ruby fluorescence in the experiments.^[48]^

*DFT calculations*: First principles simulations have been performed within the framework of density functional theory.^[49]^ The VASP package was used to carry out calculations using the pseudopotential method and the projector augmented-wave scheme (PAW).^[50-52]^ Highly accurate converged results were obtained by extending the set of plane waves up to a kinetic energy cutoff of 540 eV. The exchange-correlation energy was described using the generalized gradient approximation (GGA) according to the AM05 Armiento and Mattsson prescription.^[53]^ The AM05 exchange–correlation functional was employed because it provides reliable equilibrium structural parameters and compressibility trends for oxide framework materials. Ti is formally tetravalent (3*d*^0^) in the Na_2_TiSiO_5_ phases considered here; thus, no partially occupied Ti 3*d* states or strong correlation effects are expected, and no Hubbard U correction was applied. A dense Monkhorst-Pack grid of k-special points was used to perform integrations along the Brillouin zone (BZ) to obtain very well converged energies and forces. At each selected volume, the structure was fully relaxed to its equilibrium configuration through the calculation of the forces on atoms and the stress tensor. It should be noted that, within our simulations, the theoretical pressure, *P*(V), can be determined at the same time as the total energy, *E*(V), but independently since *P* (like other derivatives of the energy) can be obtained from the calculated stress.^[54]^ In the relaxed configurations, the forces on the atoms are lower than 0.003 eV/Å and the deviation of the stress tensor from a diagonal hydrostatic form is lower than 0.1 GPa. Born effective charges (BECs) and the dielectric constant are calculated using Density Functional Perturbation Theory (DFPT) by perturbing the system with an external electric field and analyzing the resulting changes in polarization and forces.^[55-56]^ We have performed the Bader analysis using the method developed by Henkelman et al.^[57]^ This method is efficient and robust, and provides an algorithm and tools for performing Bader's analysis on a charge density grid generated by VASP. We also obtain from our simulations the ELF for different pressures to provide insights in our study. DFT calculations were also performed using the Cambridge Sequential Total Energy Package (CASTEP) module in Material Studio to simulate Raman spectra.^[58-59]^ CASTEP was selected for these calculations because of its established phonon methodology and efficient visualization of vibrational modes. GGA in the form of PBESOL was employed and norm-conserving Pseudopotentials with a 789 eV energy cutoff were implemented.

*Estimation of effective polarization dipole* ${\overset{⃑}{p}}_{eff}$ *and phenomenological polar-coupling parameter* $\Lambda_{polar}$*:*

(1) ${\overset{⃑}{p}}_{eff}$ of the Ti-O_(nb)_ bond (taking Ti2-O_(nb)_ as an example):

Step 1: Calculate the charge center

$r_{\mathrm{center}}=\frac{Z_{Ti2,zz}^{*}\times r_{Ti2,z}+Z_{O,zz}^{*}\times r_{O,z}}{Z_{Ti2,zz}^{*}+Z_{O,zz}^{*}}$

$Z_{\mathrm{ZZ}}^{*}$ refers to the BEC tensor in direction $z$ due to atomic displacement along direction $z$;

$r$ refers to the Cartesian coordinates of the atoms in the crystal structure.

Step 2: Construct the effective polarization dipole

${\overset{⃑}{p}}_{eff}= Z_{Ti2,zz}^{*}\times{(r}_{Ti2,z}-r_{\mathrm{center}})= Z_{O,zz}^{*}\times{(r}_{O,z}-r_{\mathrm{center}})$

(2) $\Lambda_{polar}$ of Ti–O_(nb)_···Ti–O_(nb)_ chains (taking Ti2-O_(nb)_ as an example):

$\Lambda_{\mathrm{polar}}\propto\mid{\overset{⃑}{p}}_{eff}\mid^{2}/R^{3}$

$R$ refers to the distance between the dipole moments.

Calculation of the experimental mode Grüneisen parameters (γ)

$$\gamma_{{Ti-O}_{\left( nb \right)}}\left( P \right)=B\left( P \right)\times\frac{1}{\nu_{{Ti-O}_{\left( nb \right)}}}\times(\frac{d\nu_{{Ti-O}_{\left( nb \right)}}}{dP})$$

Supplementary Figures and Tables

**Guide to the Supplementary Information**

Figure S1 represents the crystal structures of Na_2_TiGeO_5_, Sr_2_TiSi_2_O_8_ and BaTiSi_2_O_8_. Figures S2–S5 provide additional evidence for the structural transition and the stability of the HP *Pca*2_1_ phase. Figures S6–S10 describe the anisotropic compressibility and structural origin of the reduced bulk modulus. Figures S11–S13 provide electronic-structure evidence for enhanced polarization and dipole–dipole interactions. Figures S14–S17 present the Raman data and mode assignments supporting the pressure-induced weakening of selected Ti–O bonds. Figures S18 and S19 provide a conceptual illustration and a comparative analysis relevant to the broader applicability of the proposed mechanism.

**Figure S1**. Crystal structures with the head-to-tail alignment of TiO_5_ square pyramids: Na_2_TiGeO_5_, Sr_2_TiSi_2_O_8_ and BaTiSi_2_O_8_. The corresponding Ti···O_(nb)_(–Ti) distances are labeled in the figure.

**Figure S2**. *In situ* powder XRD patterns of Na_2_TiSiO_5_ from 10.40 to 19.80 GPa.

**Figure S3**. Selected Le Bail and Rietveld fit plots of the *Pmc*2_1_ phase at 0.45 GPa and *Pca*2_1_ phase at 9.80 GPa, respectively. The black circles, solid red lines, solid blue lines, and orange bars represent the experimental data, simulated data, difference, and Bragg positions, respectively. Owing to the limited quality of the high-pressure powder diffraction data, only the Na atomic positions marked with asterisks in Table S1 were freely refined; the coordinates of all other atoms were fixed.

The close agreement between the Le Bail and Rietveld lattice parameters supports the reliability of the structural models used in the main text.

**Figure S4**. Symmetry breaking in the polyhedra connected along *a*-axis LP phase during the phase transition.

**Figure S5**. The comparison of powder XRD patterns of Na_2_TiSiO_5_ at 13.00 and 14.90 GPa.

The diffraction features can be indexed using the HP *Pca*2_1_ phase at both pressures. Although minor intensity variations are observed, the corresponding reflections show continuous peak shifts without the appearance of new reflections or additional peak splitting. These results indicate that no further structural transition is evident above 13.00 GPa within the investigated pressure range.

**Figure S6**. Polyhedral view of the HP phase showing the interconnected TiO_5_–SiO_4_ chains along the a′-axis and c′-axis, respectively.

**Structural Mechanism for Anisotropic Compressibility of HP Phase:**

The framework of Na_2_TiSiO_5_ consists of interconnected TiO_5_–SiO_4_ chains running along both the a′ and c′ axes (Figure S6). Under compression, the axial compressibility of such framework is typically controlled by the tilt and rotation of the polyhedrons, rather than by the compressibility of the polyhedra.^[60]^

Along the a′ axis, the TiO_5_–SiO_4_ chains extending along the a′-axis, exhibit non-uniform Ti-O-Si bond angles constrained by the *c*-slip surface. At 5.64 GPa, the bond angles are as follows: θ1 = 143.76°, θ2 =122.39°, θ3 = 124.42°, θ4 = 146.84°. Upon compression to 20.02 GPa, the bond angles are as follows: θ1 = 120.43°, θ2 =112.79°, θ3 = 143.61°, θ4 = 164.93°. These competing angular responses prevent a coherent rotational mode of the linked polyhedra. As a result, local distortions mutually compensate, leading to negligible net displacement of chain centroids along a′-axis. This suppression of cooperative tilt explains the extremely low axial compressibility (K_a_′ = 0.023(5) TPa⁻¹).

Conversely, along the c′ axis, the TiO_5_–SiO_4_ chains exhibit uniform Ti-O-Si bond angles, which is constrained by a 2_1_ screw symmetry. This necessitates a synchronized structural response across the chain units. At 5.64 GPa, the bond angles are as follows: θ1 = 137.42°, θ2 =136.70°, θ3 = 136.96°, θ4 = 137.76°. Upon compression to 20.02 GPa, the bond angles are as follows: θ1 = 118.15°, θ2 =118.06°, θ3 = 119.34°, θ4 = 120.68°. All Ti–O–Si angles decrease monotonically and synchronously under pressure, indicating a collective rotation of connected polyhedra. This cooperative tilting mechanism produces an additive projection of atomic displacements along the chain direction, leading to an efficient conversion of local angular distortion into macroscopic axial shortening. In addition, the Ti2···O_(nb)_ dipole–dipole attraction along c′-axis is equivalent to providing “additional pressure” to enhance the compression along this direction.

Therefore, the anisotropic compressibility arises from the direction-dependent cooperativity of polyhedral rotations and dipole–dipole attraction.

**Figure S7**. Compression of different BM-EOS fitting results for HP phase of Na_2_TiSiO_5_.

The comparison shows that the reduced bulk modulus of the HP phase is robust against the BM-EOS fitting choice, supporting the main-text conclusion of intrinsic compression softening.

**Figure S8**. The pressure-dependence of enthalpy difference between the HP phase and the LP phase. The blue curve represents the estimate obtained from the equation shown in the figure, whereas the red curve corresponds to the DFT calculated enthalpy difference.

The similar pressure dependence obtained from the two approaches supports the thermodynamic stabilization of the *Pca*2_1_ phase above the transition pressure, consistent with the structural transition identified from the HP-XRD measurements discussed in the main text.

**Figure S9**. Pressure-dependence of the volume of SiO_4_ tetrahedron and NaO_6_ octahedron.

Both polyhedral units show gradual volume reduction upon compression, without an anomalous expansion or abrupt change across the phase transition. This behavior indicates that the pronounced reduction in bulk modulus of the high-pressure phase is not primarily associated with anomalous compression of the SiO_4_ or NaO_6_ units, but instead is related to the pressure-induced reorganization of the Ti–O···Ti interactions discussed in the main text.

**Figure S10**. Pressure-dependence of Ti-O bond length of Na_2_TiSiO_5_ at high pressure.

**Figure S11**. Electron localization function plots of Ti1-O_(nb)_ and Ti2-O_(nb)_ cross section, which is perpendicular to the *a*-axis at 0 GPa, and to the *b’*-axis at 20 GPa. The yellow arrows indicate the weak interactions instead of chemical bonds between Ti and O.

No ELF maximum characteristic of a conventional chemical bond develops along these contacts upon compression, indicating that the shortened Ti···O_(nb)_ distances correspond to enhanced non-bonding polar interactions rather than the formation of additional Ti–O covalent bonds.

**Figure S12**. Pressure-dependence of BEC tensors perpendicular to the Ti-O_(nb)_ bonding direction.

**Figure S13**. Pressure-dependence of Bader charges for selected atoms in Na_2_TiSiO_5_. (a) Bader charges of the Ti1 and Ti2 sites. (b) Bader charges of the non-bridging oxygen atoms bonded to Ti1 and Ti2, denoted as O_nb_(–Ti1) and O_nb_(–Ti2), respectively. (c) Bader charges of the bridging oxygen atoms bonded to Ti1 and Ti2, denoted as O_b_(–Ti1) and O_b_(–Ti2), respectively. The values represent the average charges of the four bridging oxygen atoms in the TiO_5_ square pyramids.

Together, the ELF, BEC, and Bader-charge analyses distinguish pressure-enhanced dynamic polar response from simple static charge transfer, supporting the proposed dipole-mediated softening mechanism.

**Figure S14**. Selected *in situ* Raman spectra of Na_2_TiSiO_5_ in the range between 150 and 675 cm^-1^.

**Figure S15.** Assignments of selected Raman modes of LP phase Na_2_TiSiO_5_ at 0 GPa.

**Figure S16**. Assignments of selected Raman modes of HP phase Na_2_TiSiO_5_ at 8.7 GPa.

**Figure S17**. Comparison of Raman spectra of Na_2_TiSiO_5_ at 0.4 GPa (compression) and 0.5 GPa (decompression, D-0.5 GPa).

**Figure S18**. (a) Schematic illustration of bond softening and non-bonding distance reduction in Ti–O···Ti. (b) Structural analogy between Ti–O···Ti and X–H···Y.

The analogy is intended to illustrate the cooperative relationship between bond weakening and stabilization through a directional non-bonding interaction.

**Figure S19**. Reanalysis of HP-XRD data for Na_2_TiGeO_5_ from Ref. 39.^[39]^ The LP phase is stable below 11 GPa, whereas the HP phase is observed in the range of 11–20 GPa.

The reanalysis of Na_2_TiGeO_5_ provides comparative evidence that the structural mechanism proposed for Na_2_TiSiO_5_ may be transferable to related titanate frameworks, highlighting the broader relevance of dipole-mediated elastic softening.

**Table S1.** The lattice parameters of Na_2_TiSiO_5_ at high pressure

|  | LP phase  (Le Bail) | LP phase  (Rietveld) | HP phase  (Le Bail) | HP phase  (Rietveld) |
| --- | --- | --- | --- | --- |
| Space group | *Pmc2*_1_ | *Pmc2*_1_ | *Pca*2_1_ | *Pca*2_1_ |
| Pressure (GPa) | 0.45 | 0.45 | 9.80 | 9.80 |
| *a* (Å) | 9.150(12) | 9.1510(7) | 9.3278(3) | 9.3347(10) |
| *b* (Å) | 4.7821(6) | 4.7819(3) | 8.7214(6) | 8.712(2) |
| *c* (Å) | 9.8227(5) | 9.8228(3) | 9.4408(5) | 9.442(2) |
| Ti1 | 0.5, 0.4655, -0.0021 | 0.5, 0.4655, -0.0021 | 0.2643, 1.0021, 0.8314 | 0.2643, 1.0021, 0.8314 |
| Ti2 | 0, 0.0259, 0.7835 | 0, 0.0259, 0.7835 | 0.4870, 0.5122, 0.6227 | 0.4870, 0.5122, 0.6227 |
| Si1 | 0.25, 0.5043, 0.25 | 0.25, 0.5043, 0.25 | 0.2467, 0.7501, 0.0857 | 0.2467, 0.7501, 0.0857 |
| Si2 | - | - | -0.2582, 0.7539, 0.0824 | -0.2582, 0.7539, 0.0824 |
| O1 | 0.5, 0.8201, -0.002 | 0.5, 0.8201, -0.002 | 0.3186, 0.8693, -0.0184 | 0.3186, 0.8693, -0.0184 |
| O2 | 0.3519, 0.3596, 0.1334 | 0.3519, 0.3596, 0.1334 | -0.1832, 0.8357, -0.0493 | -0.1832, 0.8357, -0.0493 |
| O3 | 0.3543, 0.6452, 0.3643 | 0.3543, 0.6452, 0.3643 | 0.1829, 0.8357, 0.2161 | 0.1829, 0.8357, 0.2161 |
| O4 | 0, 0.85, 0.4454 | 0, 0.85, 0.4454 | -0.3384, 0.873, 0.1776 | -0.3384, 0.873, 0.1776 |
| O5 | 0.1472, 0.7498, 0.1836 | 0.1472, 0.7498, 0.1836 | 0.1179, 0.6611, 0.0111 | 0.1179, 0.6611, 0.0111 |
| O6 | 0.1491, 0.2598, 0.3152 | 0.1491, 0.2598, 0.3152 | -0.3748, 0.6355, 0.0249 | -0.3748, 0.6355, 0.0249 |
| O7 | - | - | 0.3642, 0.6307, 0.1379 | 0.3642, 0.6307, 0.1379 |
| O8 | - | - | -0.1322, 0.6651, 0.1648 | -0.1322, 0.6651, 0.1648 |
| O9 | - | - | 0.0825, 0.9972, 0.8319 | 0.0825, 0.9972, 0.8319 |
| O10 | - | - | 0.0700, 0.5041, 0.2897 | 0.0700, 0.5041, 0.2897 |
| Na1* | 0.5, 0.0068, 0.2544 | 0.5, -0.0582, 0.2159 | -0.5001, 0.755, 0.8537 | -0.54109, 0.7511, 0.8737 |
| Na2* | 0.2510, 0.0036, 0.0061 | 0.2628, -0.0007, 0.001 | 0.0003, 0.7516, 0.8230 | -0.0039, 0.7496, 0.7132 |
| Na3* | 0, 0.4039, 0.5040 | 0, 0.4357, 0.5055 | 0.493, 1.041, 0.0936 | 0.4882, 1.0307, 0.1116 |
| Na4* | - | - | 0.2928, 0.4987, 0.3397 | 0.2222, 0.4225, 0.0128 |

In the Rietveld refinement, only the positions of Na atoms (x, y, z) marked with asterisks were freely refined; the positions of all other atoms were fixed because the data quality was insufficient to support reliable refinement. Site occupancies were fixed to the nominal stoichiometry. Isotropic displacement parameters were fixed

**Table S2.** Calculated elastic constants *C*_ij_ for Na_2_TiSiO_5_.

| *C*_ij_ (GPa) | LP phase at 0 GPa | HP phase at 12.3 GPa |
| --- | --- | --- |
| *C*_11_ | 179.78 | 161.22 |
| *C*_22_ | 120.13 | 120.87 |
| *C*_33_ | 135.34 | 100.78 |
| *C*_44_ | 47.91 | 49.60 |
| *C*_55_ | 53.44 | 46.26 |
| *C*_66_ | 59.60 | 61.03 |
| *C*_12_ | 62.40 | 99.81 |
| *C*_13_ | 59.04 | 86.88 |
| *C*_23_ | 42.48 | 46.35 |

**Table S3.** Born effective charge tensors of Ti1, Ti2 and O_(nb)_ in Na_2_TiSiO_5_ from 0 to 20 GPa.

| Pressure / GPa | Atoms | *Zij* | | |
| --- | --- | --- | --- | --- |
| 0 | Ti1 | 4.6512 | 0.0000 | 0.0000 |
|  |  | 0.0000 | 3.0456 | -0.0810 |
|  |  | 0.0000 | 0.0428 | 4.7598 |
|  | O_(nb)_(-Ti1) | -0.9849 | 0.0000 | 0.0000 |
|  |  | 0.0000 | -2.7016 | 0.0229 |
|  |  | 0.0000 | 0.0104 | -0.9946 |
|  | Ti2 | 4.6408 | 0.0000 | 0.0000 |
|  |  | 0.0000 | 4.5157 | -0.9837 |
|  |  | 0.0000 | -0.1717 | 2.9009 |
|  | O_(nb)_(-Ti2) | -1.0284 | 0.0000 | 0.0000 |
|  |  | 0.0000 | -1.2845 | 0.5343 |
|  |  | 0.0000 | 0.4607 | -2.3457 |
| 1.0 | Ti1 | 4.6488 | 0.0000 | 0.0000 |
|  |  | 0.0000 | 3.0720 | -0.0786 |
|  |  | 0.0000 | 0.0455 | 4.7516 |
|  | O_(nb)_(-Ti1) | -0.9869 | 0.0000 | 0.0000 |
|  |  | 0.0000 | -2.7128 | 0.0220 |
|  |  | 0.0000 | 0.0096 | -0.9979 |
|  | Ti2 | 4.6414 | 0.0000 | 0.0000 |
|  |  | 0.0000 | 4.5002 | -0.9893 |
|  |  | 0.0000 | -0.1755 | 2.9247 |
|  | O_(nb)_(-Ti2) | -1.0308 | 0.0000 | 0.0000 |
|  |  | 0.0000 | -1.2876 | 0.5333 |
|  |  | 0.0000 | 0.4594 | -2.3498 |
| 2.0 | Ti1 | 4.6457 | 0.0000 | 0.0000 |
|  |  | 0.0000 | 3.0982 | -0.0750 |
|  |  | 0.0000 | 0.0501 | 4.7439 |
|  | O_(nb)_(-Ti1) | -0.9889 | 0.0000 | 0.0000 |
|  |  | 0.0000 | -2.7240 | 0.0208 |
|  |  | 0.0000 | 0.0079 | -1.0013 |
|  | Ti2 | 4.6400 | 0.0000 | 0.0000 |
|  |  | 0.0000 | 4.4846 | -0.9958 |
|  |  | 0.0000 | -0.1803 | 2.9498 |
|  | O_(nb)_(-Ti2) | -1.0331 | 0.0000 | 0.0000 |
|  |  | 0.0000 | -1.2900 | 0.5315 |
|  |  | 0.0000 | 0.4577 | -2.3538 |
| 3.2 | Ti1 | 4.6437 | 0.0000 | 0.0000 |
|  |  | 0.0000 | 3.1269 | -0.0725 |
|  |  | 0.0000 | 0.0526 | 4.7365 |
|  | O_(nb)_(-Ti1) | -0.9911 | 0.0000 | 0.0000 |
|  |  | 0.0000 | -2.7379 | 0.0197 |
|  |  | 0.0000 | 0.0070 | -1.0049 |
|  | Ti2 | 4.6428 | 0.0000 | 0.0000 |
|  |  | 0.0000 | 4.4710 | -1.0003 |
|  |  | 0.0000 | -0.1844 | 2.9752 |
|  | O_(nb)_(-Ti2) | -1.0358 | 0.0000 | 0.0000 |
|  |  | 0.0000 | -1.2926 | 0.5301 |
|  |  | 0.0000 | 0.4561 | -2.3594 |
| 4.4 | Ti1 | 4.6415 | 0.0000 | 0.0000 |
|  |  | 0.0000 | 3.1508 | -0.0698 |
|  |  | 0.0000 | 0.0559 | 4.7285 |
|  | O_(nb)_(-Ti1) | -0.9931 | 0.0000 | 0.0000 |
|  |  | 0.0000 | -2.7496 | 0.0197 |
|  |  | 0.0000 | 0.0054 | -1.0085 |
|  | Ti2 | 4.6448 | 0.0000 | 0.0000 |
|  |  | 0.0000 | 4.4572 | -1.0045 |
|  |  | 0.0000 | -0.1891 | 3.0027 |
|  | O_(nb)_(-Ti2) | -1.0384 | 0.0000 | 0.0000 |
|  |  | 0.0000 | -1.2944 | 0.5279 |
|  |  | 0.0000 | 0.4538 | -2.3662 |
| 5.7 | Ti1 | 4.6406 | 0.0000 | 0.0000 |
|  |  | 0.0000 | 3.1603 | -0.0658 |
|  |  | 0.0000 | 0.0611 | 4.7227 |
|  | O_(nb)_(-Ti1) | -0.9953 | 0.0000 | 0.0000 |
|  |  | 0.0000 | -2.7604 | 0.0187 |
|  |  | 0.0000 | 0.0036 | -1.0122 |
|  | Ti2 | 4.6451 | 0.0000 | 0.0000 |
|  |  | 0.0000 | 4.4447 | -1.0095 |
|  |  | 0.0000 | -0.1941 | 3.0303 |
|  | O_(nb)_(-Ti2) | -1.0408 | 0.0000 | 0.0000 |
|  |  | 0.0000 | -1.2952 | 0.5255 |
|  |  | 0.0000 | 0.4517 | -2.3724 |
| 6.7 | Ti1 | 3.1699 | 0.0014 | 0.0642 |
|  |  | 0.0014 | 4.6397 | -0.0726 |
|  |  | -0.0588 | 0.1543 | 4.7032 |
|  | O_(nb)_(-Ti1) | -2.7632 | 0.0004 | -0.0184 |
|  |  | 0.0015 | -1.0024 | 0.0265 |
|  |  | -0.0013 | -0.0060 | -1.0212 |
|  | Ti2 | 4.4670 | 0.0405 | 1.0010 |
|  |  | 0.0125 | 4.6531 | 0.1273 |
|  |  | 0.1815 | 0.0544 | 3.0704 |
|  | O_(nb)_(-Ti2) | -1.2893 | 0.0184 | -0.5114 |
|  |  | 0.0094 | -1.0480 | 0.0079 |
|  |  | -0.4485 | 0.0306 | -2.3881 |
| 7.5 | Ti1 | 3.1711 | 0.0033 | 0.0692 |
|  |  | 0.0073 | 4.6398 | -0.1217 |
|  |  | -0.0427 | 0.2547 | 4.6711 |
|  | O_(nb)_(-Ti1) | -2.7559 | 0.0028 | -0.0189 |
|  |  | 0.0040 | -1.0135 | 0.0439 |
|  |  | 0.0015 | -0.0095 | -1.0345 |
|  | Ti2 | 4.5156 | 0.0722 | 0.9787 |
|  |  | 0.0221 | 4.6669 | 0.2178 |
|  |  | 0.1536 | 0.0911 | 3.1202 |
|  | O_(nb)_(-Ti2) | -1.2778 | 0.0327 | -0.4881 |
|  |  | 0.0163 | -1.0591 | 0.0142 |
|  |  | -0.4413 | 0.0576 | -2.4089 |
| 8.3 | Ti1 | 3.1756 | 0.0059 | 0.0749 |
|  |  | 0.0172 | 4.6416 | -0.1535 |
|  |  | -0.0252 | 0.3144 | 4.6393 |
|  | O_(nb)_(-Ti1) | -2.7545 | 0.0063 | -0.0199 |
|  |  | 0.0071 | -1.0244 | 0.0545 |
|  |  | 0.0026 | -0.0116 | -1.0473 |
|  | Ti2 | 4.5590 | 0.0958 | 0.9560 |
|  |  | 0.0291 | 4.6791 | 0.2800 |
|  |  | 0.1270 | 0.1139 | 3.1711 |
|  | O_(nb)_(-Ti2) | -1.2658 | 0.0438 | -0.4654 |
|  |  | 0.0217 | -1.0694 | 0.0212 |
|  |  | -0.4328 | 0.0812 | -2.4309 |
| 9.2 | Ti1 | 3.1826 | 0.0090 | 0.0788 |
|  |  | 0.0277 | 4.6438 | -0.1763 |
|  |  | 0.0106 | 0.3530 | 4.6095 |
|  | O_(nb)_(-Ti1) | -2.7470 | 0.0094 | -0.0205 |
|  |  | 0.0092 | -1.0343 | 0.0617 |
|  |  | 0.0035 | -0.0127 | -1.0592 |
|  | Ti2 | 4.5954 | 0.1160 | 0.9347 |
|  |  | 0.0356 | 4.6892 | 0.3272 |
|  |  | 0.1024 | 0.1307 | 3.2209 |
|  | O_(nb)_(-Ti2) | -1.2553 | 0.0528 | -0.4441 |
|  |  | 0.0265 | -1.0788 | 0.0275 |
|  |  | 0.4248 | 0.1025 | -2.4512 |
| 10.1 | Ti1 | 3.1893 | 0.0150 | 0.0860 |
|  |  | 0.0474 | 4.6475 | -0.2004 |
|  |  | 0.0124 | 0.3852 | 4.5766 |
|  | O_(nb)_(-Ti1) | -2.7422 | 0.0154 | -0.0216 |
|  |  | 0.0135 | -1.0456 | 0.0676 |
|  |  | 0.0034 | -0.0145 | -1.0725 |
|  | Ti2 | 4.6356 | 0.1377 | 0.9081 |
|  |  | 0.0431 | 4.6981 | 0.3755 |
|  |  | 0.0747 | 0.1438 | 3.2756 |
|  | O_(nb)_(-Ti2) | -1.2415 | 0.0647 | -0.4192 |
|  |  | 0.0326 | -1.0880 | 0.0363 |
|  |  | -0.4125 | 0.1296 | -2.4736 |
| 11.2 | Ti1 | 3.2018 | 0.0208 | 0.0917 |
|  |  | 0.0651 | 4.6494 | -0.2182 |
|  |  | 0.0320 | 0.3999 | 4.5513 |
|  | O_(nb)_(-Ti1) | -2.7406 | 0.0206 | -0.0227 |
|  |  | 0.0161 | -1.0546 | 0.0712 |
|  |  | 0.0030 | -0.0159 | -1.0846 |
|  | Ti2 | 4.6636 | 0.1549 | 0.8860 |
|  |  | 0.0509 | 4.6975 | 0.4068 |
|  |  | 0.0533 | 0.1516 | 3.3290 |
|  | O_(nb)_(-Ti2) | -1.2302 | 0.0743 | -0.3984 |
|  |  | 0.0380 | -1.0951 | 0.0453 |
|  |  | -0.4012 | 0.1533 | -2.4939 |
| 12.3 | Ti1 | 3.2156 | 0.0293 | 0.0979 |
|  |  | 0.0884 | 4.6515 | -0.2339 |
|  |  | 0.0532 | 0.4094 | 4.5287 |
|  | O_(nb)_(-Ti1) | -2.7407 | 0.0265 | -0.0242 |
|  |  | 0.0181 | -1.0641 | 0.0739 |
|  |  | 0.0015 | -0.0175 | -1.0971 |
|  | Ti2 | 4.6904 | 0.1727 | 0.8653 |
|  |  | 0.0598 | 4.6967 | 0.4376 |
|  |  | 0.0328 | 0.1567 | 3.3851 |
|  | O_(nb)_(-Ti2) | -1.2182 | 0.0849 | -0.3768 |
|  |  | 0.0448 | -1.1014 | 0.0563 |
|  |  | -0.3886 | 0.1793 | -2.5156 |
| 13.6 | Ti1 | 3.2320 | 0.0396 | 0.1050 |
|  |  | 0.1166 | 4.6524 | -0.2483 |
|  |  | 0.0772 | 0.4120 | 4.5097 |
|  | O_(nb)_(-Ti1) | -2.7426 | 0.0340 | -0.0263 |
|  |  | 0.0197 | -1.0737 | 0.0756 |
|  |  | -0.0003 | -0.0196 | -1.1097 |
|  | Ti2 | 4.7141 | 0.1918 | 0.8441 |
|  |  | 0.0680 | 4.6913 | 0.4690 |
|  |  | 0.0128 | 0.1585 | 3.4416 |
|  | O_(nb)_(-Ti2) | -1.2066 | 0.0970 | -0.3550 |
|  |  | 0.0529 | -1.1065 | 0.0688 |
|  |  | -0.3756 | 0.2072 | -2.5361 |
| 15.0 | Ti1 | 3.2346 | 0.0498 | 0.1099 |
|  |  | 0.1419 | 4.6518 | -0.2593 |
|  |  | 0.0975 | 0.4099 | 4.4965 |
|  | O_(nb)_(-Ti1) | -2.7420 | 0.0412 | -0.0287 |
|  |  | 0.0202 | -1.0825 | 0.0764 |
|  |  | -0.0017 | -0.0218 | -1.1220 |
|  | Ti2 | 4.7330 | 0.2123 | 0.8244 |
|  |  | 0.0773 | 4.6837 | 0.4957 |
|  |  | -0.0045 | 0.1579 | 3.5004 |
|  | O_(nb)_(-Ti2) | -1.1952 | 0.1087 | -0.3344 |
|  |  | 0.0614 | -1.1107 | 0.0814 |
|  |  | -0.3637 | 0.2340 | -2.5572 |
| 16. | Ti1 | 3.2610 | 0.0605 | 0.1134 |
|  |  | 0.1681 | 4.6512 | -0.2679 |
|  |  | 0.1148 | 0.4052 | 4.4880 |
|  | O_(nb)_(-Ti1) | -2.7449 | 0.0489 | -0.0313 |
|  |  | 0.0188 | -1.0911 | 0.0767 |
|  |  | -0.0027 | -0.0241 | -1.1342 |
|  | Ti2 | 4.7474 | 0.2362 | 0.8052 |
|  |  | 0.0880 | 4.6740 | 0.5193 |
|  |  | -0.0202 | 0.1545 | 3.5618 |
|  | O_(nb)_(-Ti2) | -1.1859 | 0.1208 | -0.3127 |
|  |  | 0.0703 | -1.1141 | 0.0931 |
|  |  | -0.3529 | 0.2614 | -2.5810 |
| 18.2 | Ti1 | 3.2694 | 0.0664 | 0.1127 |
|  |  | 0.1844 | 4.6525 | -0.2710 |
|  |  | 0.1214 | 0.3986 | 4.4875 |
|  | O_(nb)_(-Ti1) | -2.7476 | 0.0535 | -0.0328 |
|  |  | 0.0158 | -1.0982 | 0.0774 |
|  |  | -0.0010 | -0.0255 | -1.1473 |
|  | Ti2 | 4.7587 | 0.2556 | 0.7935 |
|  |  | 0.0971 | 4.6724 | 0.5315 |
|  |  | -0.0312 | 0.1495 | 3.6279 |
|  | O_(nb)_(-Ti2) | -1.1788 | 0.1279 | -0.2944 |
|  |  | 0.0765 | -1.1187 | 0.1011 |
|  |  | -0.3456 | 0.2834 | -2.6093 |
| 20.0 | Ti1 | 3.2925 | 0.0705 | 0.1103 |
|  |  | 0.1903 | 4.6550 | -0.2700 |
|  |  | 0.1193 | 0.3914 | 4.4945 |
|  | O_(nb)_(-Ti1) | -2.7503 | 0.0546 | -0.0335 |
|  |  | 0.0119 | -1.1039 | 0.0786 |
|  |  | 0.0024 | -0.0264 | -1.1622 |
|  | Ti2 | 4.7688 | 0.2685 | 0.7892 |
|  |  | 0.1037 | 4.6809 | 0.5327 |
|  |  | -0.0382 | 0.1428 | 3.7009 |
|  | O_(nb)_(-Ti2) | -1.1754 | 0.1300 | -0.2787 |
|  |  | 0.0800 | -1.1246 | 0.1055 |
|  |  | -0.3404 | 0.3021 | -2.6447 |

**Table S4.** The magnitude of the dipole moments ${\overset{⃑}{p}}_{eff}$ of Ti1-O_(nb)_ at different pressures

| *P* / GPa | $Z_{Ti1,yy}^{*}$ (LP)  $Z_{Ti1,xx}^{*}$ (HP) | $r_{y}$ (LP) or  $r_{x}$ (HP) / Å | $Z_{O_{(nb)},yy}^{*}$ (LP)  $Z_{O_{(nb)},xx}^{*}$ (HP) | $r_{y}$ (LP) or  $r_{x}$ (HP) / Å | $r_{center}$ / Å | \|${\overset{⃑}{p}}_{\mathrm{eff}}$\| / D |
| --- | --- | --- | --- | --- | --- | --- |
| 0 | 3.04564 | 2.26343 | -2.70159 | 3.98045 | 3.12194 | 12.54182 |
| 1.0 | 3.07202 | 2.24458 | -2.71277 | 3.96154 | 3.10306 | 12.65003 |
| 2.0 | 3.09816 | 2.22593 | -2.72396 | 3.94275 | 3.08434 | 12.75662 |
| 3.2 | 3.12689 | 2.20776 | -2.73787 | 3.92472 | 3.06624 | 12.87596 |
| 4.4 | 3.15078 | 2.19016 | -2.74963 | 3.90710 | 3.04863 | 12.97419 |
| 5.7 | 3.16027 | 2.17093 | -2.76042 | 3.88755 | 3.02924 | 13.01084 |
| 6.7 | 3.16994 | 2.17211 | -2.76316 | 3.88905 | 3.03058 | 13.05310 |
| 7.5 | 3.17107 | 2.18874 | -2.75593 | 3.90585 | 3.04730 | 13.05904 |
| 8.3 | 3.17562 | 2.20269 | -2.75445 | 3.92009 | 3.06139 | 13.07996 |
| 9.2 | 3.18263 | 2.21309 | -2.74695 | 3.93033 | 3.07171 | 13.10763 |
| 10.1 | 3.18929 | 2.22749 | -2.74218 | 3.94450 | 3.08600 | 13.13330 |
| 11.2 | 3.20183 | 2.23513 | -2.74061 | 3.95179 | 3.09346 | 13.18227 |
| 12.3 | 3.21556 | 2.24315 | -2.74070 | 3.95932 | 3.10124 | 13.23499 |
| 13.6 | 3.23196 | 2.25052 | -2.74262 | 3.96605 | 3.10829 | 13.29753 |
| 15.0 | 3.23458 | 2.25587 | -2.74200 | 3.97061 | 3.11324 | 13.30219 |
| 16.5 | 3.26096 | 2.26041 | -2.74489 | 3.97411 | 3.11726 | 13.40253 |
| 18.2 | 3.26936 | 2.26083 | -2.74762 | 3.97369 | 3.11726 | 13.43050 |
| 20.0 | 3.29249 | 2.25813 | -2.75028 | 3.97004 | 3.11409 | 13.51799 |

${\overset{⃑}{p}}_{eff}= Z_{Ti1,zz}^{*}\times{(r}_{Ti1,z}-r_{\mathrm{center}})= Z_{O,zz}^{*}\times{(r}_{O,z}-r_{\mathrm{center}})$

**Table S5.** The magnitude of the dipole moments ${\overset{⃑}{p}}_{eff}$ of Ti2-O_(nb)_ at different pressures

| *P* / GPa | $Z_{Ti2,zz}^{*}$ | $r_{z}$ / Å | $Z_{O_{(nb)},zz}^{*}$ | $r_{z}$ / Å | $r_{center}$ / Å | \|${\overset{⃑}{p}}_{\mathrm{eff}}$\| / D |
| --- | --- | --- | --- | --- | --- | --- |
| 0 | 2.9009 | 2.83521 | -2.34574 | 4.43956 | 3.637385 | 11.16193 |
| 1.0 | 2.92467 | 2.82382 | -2.34984 | 4.42743 | 3.625625 | 11.24822 |
| 2.0 | 2.94984 | 2.81259 | -2.35378 | 4.41569 | 3.61414 | 11.34137 |
| 3.2 | 2.97525 | 2.80135 | -2.35944 | 4.40404 | 3.602695 | 11.43615 |
| 4.4 | 3.00269 | 2.78978 | -2.36625 | 4.39251 | 3.591145 | 11.54192 |
| 5.7 | 3.0303 | 2.77894 | -2.37243 | 4.38174 | 3.58034 | 11.64854 |
| 6.7 | 3.07038 | 1.16549 | -2.38806 | 2.77004 | 1.967765 | 11.81552 |
| 7.5 | 3.12023 | 1.15647 | -2.40893 | 2.76455 | 1.96051 | 12.03376 |
| 8.3 | 3.17108 | 1.14726 | -2.43087 | 2.75873 | 1.952995 | 12.25566 |
| 9.2 | 3.22094 | 1.13846 | -2.45118 | 2.75246 | 1.94546 | 12.46791 |
| 10.1 | 3.27564 | 1.12743 | -2.47359 | 2.7443 | 1.935865 | 12.70217 |
| 11.2 | 3.32903 | 1.11687 | -2.49391 | 2.73554 | 1.926205 | 12.92359 |
| 12.3 | 3.38507 | 1.10492 | -2.51563 | 2.72518 | 1.91505 | 13.15407 |
| 13.6 | 3.44165 | 1.09149 | -2.53609 | 2.7121 | 1.901795 | 13.37682 |
| 15.0 | 3.50036 | 1.07723 | -2.55721 | 2.69774 | 1.887485 | 13.60413 |
| 16.5 | 3.56183 | 1.06175 | -2.58103 | 2.68188 | 1.871815 | 13.83979 |
| 18.2 | 3.62793 | 1.04765 | -2.60934 | 2.6676 | 1.857625 | 14.09507 |
| 20.0 | 3.70095 | 1.03452 | -2.64466 | 2.65481 | 1.844665 | 14.38181 |

${\overset{⃑}{p}}_{eff}= Z_{Ti2,zz}^{*}\times{(r}_{Ti2,z}-r_{\mathrm{center}})= Z_{O,zz}^{*}\times{(r}_{O,z}-r_{\mathrm{center}})$

**Table S6.** The polar-coupling parameter $\Lambda_{\mathrm{polar}}$ of dipole-dipole interactions of Ti1-O_(nb)_···Ti1-O_(nb)_ and Ti2-O_(nb)_···Ti2-O_(nb)_ at different pressures

| *P* / GPa | Ti1-O_(nb)_···Ti1-O_(nb)_ | | | Ti2-O_(nb)_···Ti2-O_(nb)_ | | |
| --- | --- | --- | --- | --- | --- | --- |
|  | \|${\overset{⃑}{p}}_{eff}$\| / D | $R$ / Å | $\Lambda_{\mathrm{polar}}$ | \|${\overset{⃑}{p}}_{eff}$\| / D | $R$ / Å | $\Lambda_{\mathrm{polar}}$ |
| 0 | 12.54182 | 4.83504 | -1.39162 | 11.16193 | 4.954755 | -1.02426 |
| 1.0 | 12.65003 | 4.80968 | -1.43825 | 11.24822 | 4.93329 | -1.0538 |
| 2.0 | 12.75662 | 4.78489 | -1.48544 | 11.34137 | 4.911785 | -1.08546 |
| 3.2 | 12.87596 | 4.75943 | -1.53778 | 11.43615 | 4.89045 | -1.11818 |
| 4.4 | 12.97419 | 4.73375 | -1.58688 | 11.54192 | 4.86856 | -1.1544 |
| 5.7 | 13.01084 | 4.70825 | -1.62193 | 11.64854 | 4.84727 | -1.19138 |
| 6.7 | 13.05310 | 4.70214 | -1.63886 | 11.81552 | 4.818475 | -1.24789 |
| 7.5 | 13.05904 | 4.712435 | -1.62962 | 12.03376 | 4.783945 | -1.32265 |
| 8.3 | 13.07996 | 4.72073 | -1.62624 | 12.25566 | 4.749385 | -1.40204 |
| 9.2 | 13.10763 | 4.725805 | -1.62787 | 12.46791 | 4.715695 | -1.48235 |
| 10.1 | 13.13330 | 4.734505 | -1.62526 | 12.70217 | 4.680075 | -1.57397 |
| 11.2 | 13.18227 | 4.73665 | -1.63518 | 12.92359 | 4.64548 | -1.666 |
| 12.3 | 13.23499 | 4.73898 | -1.64586 | 13.15407 | 4.610355 | -1.7657 |
| 13.6 | 13.29753 | 4.73954 | -1.66086 | 13.37682 | 4.57569 | -1.86782 |
| 15.0 | 13.30219 | 4.73764 | -1.66403 | 13.60413 | 4.541045 | -1.9764 |
| 16.5 | 13.40253 | 4.734235 | -1.69287 | 13.83979 | 4.5066 | -2.09272 |
| 18.2 | 13.43050 | 4.728785 | -1.70583 | 14.09507 | 4.47028 | -2.22398 |
| 20.0 | 13.51799 | 4.72253 | -1.735 | 14.38181 | 4.43089 | -2.37768 |

$\Lambda_{\mathrm{polar}}\propto\mid{\overset{⃑}{p}}_{eff}\mid^{2}/R^{3}$

**Table S7.** The mode Grüneisen parameters of Na_2_TiSiO_5_ at high pressure

| P / GPa | *B*_0_ / GPa | *B*(*P*) / GPa | ν(Ti1–O_(nb)_) / cm^-1^ | dν/dP (Ti1) / cm^-1^⋅ GPa | γ (Ti1) | ν(Ti2–O_(nb)_) / cm^-1^ | dν/dP (Ti2) / cm^-1^⋅ GPa | γ (Ti2) |
| --- | --- | --- | --- | --- | --- | --- | --- | --- |
| 0.4 | 94 | 95.6 | 832.88 | 0.73 | 0.08 | 855.14 | 1.39 | 0.16 |
| 8.7 | 55.4 | 90.2 | 840.24 | 0.95 | 0.10 | 857.25 | -0.8 | -0.08 |

$\gamma_{{Ti-O}_{\left( nb \right)}}\left( P \right)=B\left( P \right)\times\frac{1}{\nu_{{Ti-O}_{\left( nb \right)}}}\times(\frac{d\nu_{{Ti-O}_{\left( nb \right)}}}{dP})$

References

[40] A. Ziadi, H. Hillebrecht, G. Thiele, B. Elouadi, “Crystal structure of orthorhombic LT-Na_2_TiSiO_5_ and its relation to the tetragonal HT-form,” *Journal of Solid State Chemistry* **123**, no. 2 (1996): 324–330.
[41] J. Ding, Y. Li, Q. Wu, Q. Long, Y. Wang, Y. Wang, “A novel self-activated white-light-emitting phosphor of Na_2_TiSiO_5_ with two Ti sites of TiO_5_ and TiO_6_,” *RSC Advances* **6**, no. 11 (2016): 8605–8611.
[42] S. Klotz, J.-C. Chervin, P. Munsch, G. Le Marchand, “Hydrostatic limits of 11 pressure transmitting media,” *Journal of Physics D: Applied Physics* **42**, no. 7 (2009): 075413.
[43] A. Dewaele, P. Loubeyre, M. Mezouar, “Equations of state of six metals above 94 GPa,” *Physical Review B* **70**, no. 9 (2004): 094112.
[44] F. Fauth, I. Peral, C. Popescu, M. Knapp, “The new material science powder diffraction beamline at ALBA synchrotron,” *Powder Diffraction* **28**, Suppl. S2 (2013): S360–S370.
[45] C. Prescher, V. B. Prakapenka, “DIOPTAS: a program for reduction of two-dimensional X-ray diffraction data and data exploration,” *High Pressure Research* **35**, no. 3 (2015): 223–230.
[46] V. Petříček, M. Dušek, L. Palatinus, “Crystallographic computing system JANA2006: general features,” *Zeitschrift für Kristallographie – Crystalline Materials* **229**, no. 5 (2014): 345–352.
[47] W. Kraus, G. Nolze, “POWDER CELL – a program for the representation and manipulation of crystal structures and calculation of the resulting X-ray powder patterns,” *Journal of Applied Crystallography* **29**, no. 3 (1996): 301–303.
[48] H. K. Mao, J. Xu, P. M. Bell, “Calibration of the ruby pressure gauge to 800 kbar under quasi-hydrostatic conditions,” *Journal of Geophysical Research: Solid Earth* **91**, no. B5 (1986): 4673–4676.
[49] P. Hohenberg, W. Kohn, “Inhomogeneous electron gas,” *Physical Review* **136**, no. 3B (1964): B864–B871.
[50] G. Kresse, J. Furthmüller, “Efficient iterative schemes for *ab initio* total-energy calculations using a plane-wave basis set,” *Physical Review B* **54**, no. 16 (1996): 11169–11186.
[51] P. E. Blöchl, “Projector augmented-wave method,” *Physical Review B* **50**, no. 24 (1994): 17953–17979.
[52] G. Kresse, D. Joubert, “From ultrasoft pseudopotentials to the projector augmented-wave method,” *Physical Review B* **59**, no. 3 (1999): 1758–1775.
[53] A. E. Mattsson, R. Armiento, J. Paier, G. Kresse, J. M. Wills, T. R. Mattsson, “The AM05 density functional applied to solids,” *The Journal of Chemical Physics* **128**, no. 8 (2008): 084714.
[54] A. Mujica, A. Rubio, A. Muñoz, R. J. Needs, “High-pressure phases of group-IV, III–V, and II–VI compounds,” *Reviews of Modern Physics* **75**, no. 3 (2003): 863–912.
[55] X. Gonze, C. Lee, “Dynamical matrices, Born effective charges, dielectric permittivity tensors, and interatomic force constants from density-functional perturbation theory,” *Physical Review B* **55**, no. 16 (1997): 10355–10368.
[56] M. Gajdoš, K. Hummer, G. Kresse, J. Furthmüller, F. Bechstedt, “Linear optical properties in the projector-augmented wave methodology,” *Physical Review B* **73**, no. 4 (2006): 045112.
[57] G. Henkelman, A. Arnaldsson, H. Jónsson, “A fast and robust algorithm for Bader decomposition of charge density,” *Computational Materials Science* **36**, no. 3 (2006): 254–360.
[58] S. J. Clark, M. D. Segall, C. J. Pickard, *et al.*, “First principles methods using CASTEP,” *Zeitschrift für Kristallographie – Crystalline Materials* **220**, no. 5–6 (2005): 567–570.
[59] S. Baroni, S. de Gironcoli, A. Dal Corso, P. Giannozzi, “Phonons and related crystal properties from density-functional perturbation theory,” *Reviews of Modern Physics* 73, no. 2 (2001): 515–562.
[60] R. J. Angel, N. L. Ross, J. Zhao, “The compression of framework minerals: beyond rigid polyhedra,” *European Journal of Mineralogy* **17**, no. 2, (2005): 193–200.
